# Extracting the characteristics of life cycle assessments via data mining

**DOI:** 10.1016/j.mex.2020.101004

**Published:** 2020-07-22

**Authors:** Nancy Diaz-Elsayed, Qiong Zhang

**Affiliations:** Department of Civil & Environmental Engineering, University of South Florida, 4202 E. Fowler Ave, ENG 030, Tampa, FL 33620, USA

**Keywords:** Data mining, Life cycle assessment, Natural Language Toolkit (NLTK), Regular expressions

## Abstract

Life cycle assessments (LCAs) follow the ISO 14040 standard and consist of the following steps: 1) goal and scope definition, 2) life cycle inventory analysis, 3) life cycle impact assessment, and 4) interpretation. Prior literature reviews of wastewater treatment and water reuse LCAs have evaluated the methods implemented within these assessments. In lieu of manually tabulating the characteristic features of LCAs, Data Mining LCAs provides a method to facilitate the extraction of key characteristics. The process consists of the following:

• Each journal article is converted to a text file and read in Python.
• Search terms are defined for each characteristic of the LCA to be extracted.
• By employing Python's regular expressions operations and the natural language toolkit (NLTK), the functional unit, life cycle impact characterization method, and the location of each case study are identified.

## Graphical_Abstract

**Figure:**
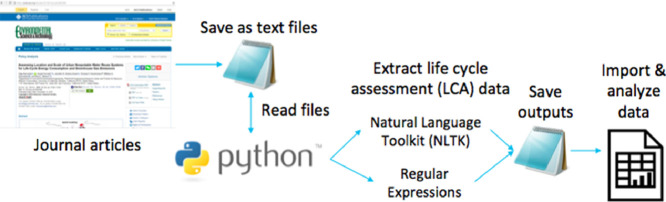

**Table:** Specifications Table

Subject Area:	Engineering
More specific subject area:	Life Cycle Assessments
Method name:	Data Mining Life Cycle Assessments (Data Mining LCAs)
Name and reference of original method:	Not Applicable
Resource availability:	https://www.anaconda.com/distribution/

## Method details

### Overview

Prior reviews concerning the life cycle assessment (LCA) of wastewater treatment, water reuse, and biosolids management schemes have collected information about the methods that were implemented [1], [2], [3], [4]. This is feasible because LCAs typically follow the ISO 14040 standard [5], which describes the steps to conduct an LCA as follows: 1) goal and scope definition, 2) life cycle inventory analysis, 3) life cycle impact assessment, and 4) interpretation. The conventional means of conducting these literature reviews is by reading each article and tabulating each key aspect of the life cycle assessment (e.g., the functional unit or the characterization method used for the life cycle impact assessment); the search function can also be used to speed up the process. Since extracting such characteristics from each life cycle assessment is a repetitive process, a more efficient, automated method of extracting this data was developed: Data Mining Life Cycle Assessments (Data Mining LCAs). Our method leverages Python, the regular expressions operations, and the machine learning elements embedded in the natural language toolkit (NLTK).

The following steps were employed and the details concerning each step will be described in the sections that follow:

1) Transfer the text of the journal articles to text files,
2) Identify the functional unit used in the studies,
3) Identify the characterization methods employed for the life cycle impact assessment, and
4) Identify the location referenced in the case studies.

The method was implemented on a literature review of wastewater-based resource recovery systems. Specifically, the recovery of water, energy, and/or nutrients from wastewater was considered. The process in its entirety was done in three iterations, that is, separately for LCAs concerning water reuse, energy recovery, and nutrient recovery.

### Creating the text files

The text within the journal articles was considered as the data for this approach. Once the set of journal articles to be included in the literature review was defined for each type of resource to be recovered (i.e., water, energy, and nutrients), the text within each article was transferred to a distinct text document in Microsoft Notepad or TextEdit (for macOS). The majority of the journal articles were available online in HTML format, but a few articles were only available in PDF. For articles that are only available as a PDF file, it is recommended to copy blocks of text from the PDF files to the text file either sections or paragraphs at a time to avoid transferring information from the headers and footers (e.g., the journal title, volume number, issue number, and pagination) with the main text. One text file was created for each journal article identified for the literature review. The text files were named “PaperX.txt”, where *X* represents a number designated to the article from 1 to *N* (the total number of articles). The text files were saved in the same directory as the Python files.

Note that the programs were tested with text files that were saved with UTF-8 and Western (Windows Latin 1) encoding. The programs did not work with UTF-16 encoding, so the data mining programs would have to be modified to accommodate such encoding. Moreover, the file name of each article should include “.txt” (i.e., the first article appeared as “Paper1.txt”), so that it can be identified during the search process. FU_regex.py (available in the Supplementary Material) was tested on text files where “.txt” was not a part of the file name (i.e., “Paper1”) and although they were saved as text files, the functional unit was not extracted from these files. Therefore, “.txt” should be included in the file name or the search term should be modified in the data mining programs as appropriate.

### Identifying the functional unit

**Fig. 1:**
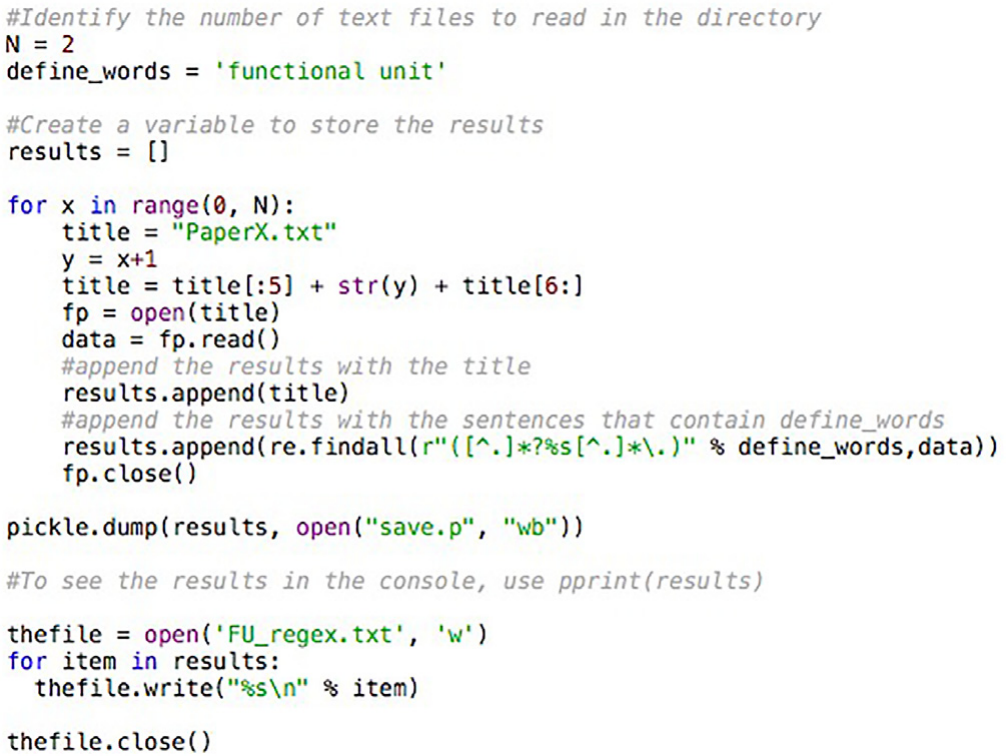
An excerpt of the code used to extract phrases containing “functional unit” based on regular expressions operations.

Statements that contained the phrase “functional unit” were extracted from the text files using the code shown in Fig. 1 (see FU_regex.py in the Supplementary Material for the entire program).

This code relies on python's regular expressions operations, where *N* represents the number of text files to read, “define_words” was set to “functional unit”, and “data” represents the read text file. The statements containing the phrase “functional unit” were saved in a text file. The text file was imported into a spreadsheet where the type of functional unit used (e.g., m^3^ of wastewater or kg of dry solids) was tabulated.

**Fig. 2:**
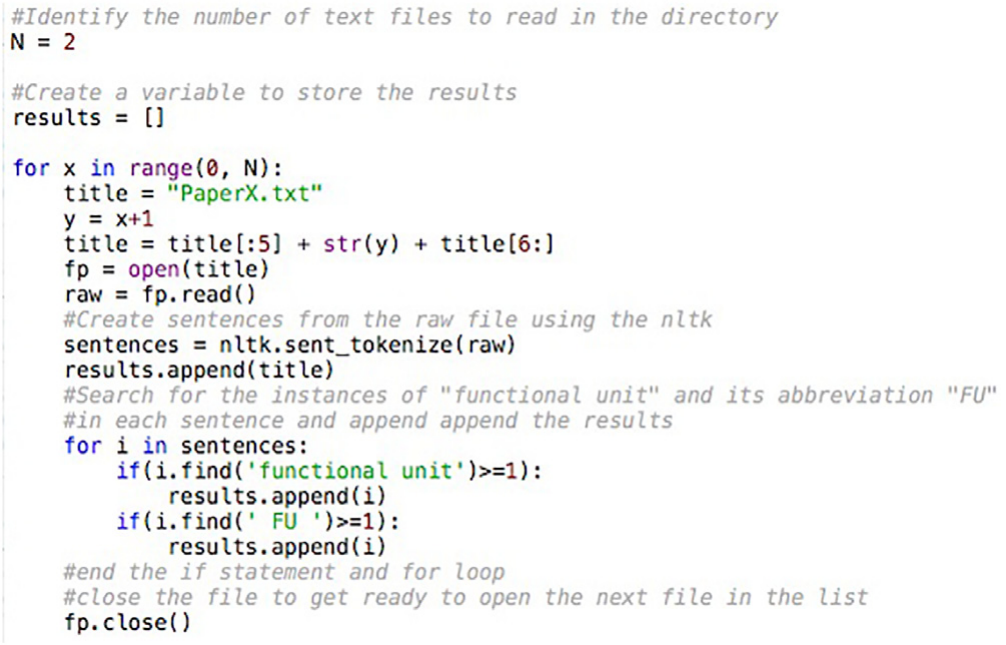
An excerpt of the code used to extract phrases containing “functional unit” or “FU” based on the Natural Language Toolkit (NLTK).

Using the “findall” operation described previously, some statements were returned that were incomplete. For example, when “i.e.” was used in a sentence that contained “functional unit”, the sentence could be extracted after “i.” from “i.e.,” instead of completing the sentence. Since the NLTK can tokenize words and sentences within a text file very effectively, the data collected with the regular expressions operations was then supplemented by the NLTK to account for instances when “functional unit” and its abbreviation “FU” was used (see Fig. 2 and FU_NLTK.py in the Supplementary Material).

### Identifying the characterization method

The characterization methods used for the life cycle impact assessment (LCIA) were then identified. The words within the text files were converted into tokens with the NLTK (see LCIA.py in the Supplementary Material), so that the frequency of use of a single word or abbreviation could be counted. A combination of NLTK and regular expressions operations was used depending on if a single word or abbreviation was being searched for (in which case the NLTK was used) or if a phrase was being searched for (in which case the “findall” regular expressions operations was used). The number of instances that a characterization method was mentioned in the text was used to deduce which method was implemented in the LCA. For instances where several characterization methods were mentioned, it was assumed that the method that was mentioned most frequently was the method implemented in the article, and the method employed was confirmed by reading the journal article directly. Alternatively, the statement containing the name of the characterization method could be mined from the text file using regular expressions operations and/or NLTK for confirmation (i.e., employing the methods used to identify the functional unit described previously). The following impact characterization methods were considered for this step of the process:

• CML
• Eco-Indicator
• Environmental Development of Industrial Products (EDIP)
• Economic Input Output-Life Cycle Assessment (EIO-LCA)
• IMPACT2002+
• ReCiPe
• Sludge land application (SLAtox)
• Tool for the Reduction and Assessment of Chemicals and Other Environmental Impacts (TRACI)
• Water-Energy Sustainability Tool (WEST)
• Wastewater-Energy Sustainability Tool (WWEST)
• USEtox

Additionally, the number of instances that “IPCC” (i.e., the Intergovernmental Panel on Climate Change) or “ILCD” (International Reference Life Cycle Data System) was mentioned was recorded, since guidelines developed by the IPCC and within the ILCD are often used in LCAs.

### Identifying the case study location

The case study location was also deduced by evaluating the number of instances that a location was mentioned using the “findall” and “FreqDist” operations (see Location.py in the Supplementary Material). Initially, the focus was on the country mentioned in the text files. That is, the number of times that a country was mentioned within each case study was counted. For papers that mentioned several countries, the case study location was confirmed by reviewing the article directly. However, since some countries were mentioned with reference to other case studies in the literature review or for the justification of the methods implemented, additional search terms were incorporated such as major cities (i.e., Sydney) in addition to prominent water reuse states from the United States (e.g., Arizona, California, Florida, and Texas). If a location was not immediately apparent using these methods, the location was identified by referring to the journal article directly, and the search methods were then modified to include relevant variations in the text (e.g., adding search terms for “Bolivia's” and “Bolivian” to be included in addition to “Bolivia”). The list of locations was sourced from [6], which was amended to add Hong Kong and Palestine.

In order to provide the context for how the names of these locations appear in the text, a similar search process as what was used to extract the statements containing the phrase “functional unit” can be used. The same could be applied for the context surrounding each characterization method considering that sometimes a characterization method is mentioned in the text to report what other studies have accomplished.

## Method Validation

For the purpose of validating the method, the programs were tested on two sample text files (see Paper1.txt and Paper 2.txt in the Supplementary Material). Table 1 shows the raw and processed outputs from running each data extraction program. Note that the first ([‘start’) and last (‘end’]) entries for these outputs were merely buffers to simplify the import process into a spreadsheet (i.e., to easily align the outputs with each category). By comparing the processed outputs to the sample text files, the validity of the extracted data can be confirmed.

**Table 1:** Outputs for the sample text files: Paper1.txt and Paper2.txt.

Feature (Program Name)	Raw Outputs	Processed Outputs
Functional Unit – NLTK (FU_NLTK.py)	Paper1.txt The functional unit implemented in this evaluation was 1 m^3 of wastewater treated, i.e., it is based on volume. Paper2.txt The functional unit (FU) implemented in this evaluation was 1 dry ton of biosolids. This FU was used to account for the dry weight of the biosolids.	Paper1: 1 m^3 of wastewater treated Paper2: 1 dry ton of biosolids
Functional Unit – Regex (FU_regex.py)	Paper1.txt [' The functional unit implemented in this evaluation was 1 m^3 of wastewater treated, i.'] Paper2.txt [' The functional unit (FU) implemented in this evaluation was 1 dry ton of biosolids.']	Paper1: 1 m^3 of wastewater treated Paper2: 1 dry ton of biosolids
Characterization Method (LCIA.py)	[‘start’, 1, 0, 0, 0, 0, 0, 0, 0, 0, 0, 0, 0, 0, 0, ‘end’] [‘start’, 0, 0, 0, 0, 0, 2, 0, 0, 1, 0, 0, 0, 0, 0, ‘end’]	Paper1: CML Paper2: IPCC & ReCiPe
Case Study Location (Location.py)	['start', 0, 0, 0, 0, 0, 0, 0, 0, 0, 0, 0, 0, 0, 0, 0, 0, 0, 0, 0, 0, 0, 0, 0, 0, 0, 0, 0, 0, 0, 0, 0, 0, 0, 0, 0, 0, 0, 0, 0, 0, 0, 0, 0, 0, 0, 0, 0, 0, 0, 0, 0, 0, 0, 0, 0, 0, 0, 0, 0, 0, 0, 0, 0, 0, 0, 0, 0, 0, 0, 0, 0, 0, 0, 0, 0, 0, 0, 0, 0, 0, 0, 0, 0, 0, 0, 0, 0, 0, 0, 0, 0, 0, 0, 0, 0, 0, 0, 0, 0, 0, 0, 0, 0, 0, 0, 0, 0, 0, 0, 0, 0, 0, 0, 0, 1, 0, 0, 0, 0, 0, 0, 0, 0, 0, 0, 0, 0, 0, 0, 0, 0, 0, 0, 0, 0, 0, 0, 0, 0, 0, 0, 0, 0, 0, 0, 0, 0, 0, 0, 0, 0, 0, 0, 0, 0, 0, 0, 0, 0, 0, 0, 0, 0, 0, 0, 0, 0, 0, 0, 0, 0, 0, 0, 0, 0, 0, 0, 0, 0, 0, 0, 0, 0, 0, 0, 0, 0, 0, 0, 0, 0, 0, 0, 0, 0, 0, 0, 'end'] ['start', 0, 0, 0, 0, 0, 0, 0, 0, 0, 0, 0, 0, 0, 0, 0, 0, 0, 0, 0, 0, 0, 0, 0, 0, 0, 0, 0, 0, 0, 0, 0, 0, 0, 0, 0, 0, 0, 0, 0, 0, 0, 0, 0, 0, 0, 0, 0, 0, 0, 0, 0, 0, 1, 0, 0, 0, 0, 0, 0, 0, 0, 0, 0, 0, 0, 0, 0, 0, 0, 0, 0, 0, 0, 0, 0, 0, 0, 0, 0, 0, 0, 0, 0, 0, 0, 0, 0, 0, 0, 0, 0, 0, 0, 0, 0, 0, 0, 0, 0, 0, 0, 0, 0, 0, 0, 0, 0, 0, 0, 0, 0, 0, 0, 0, 0, 0, 0, 0, 0, 0, 0, 0, 0, 0, 0, 0, 0, 0, 0, 0, 0, 0, 0, 0, 0, 0, 0, 0, 0, 0, 0, 0, 0, 0, 0, 0, 0, 0, 0, 0, 0, 0, 0, 0, 0, 0, 0, 0, 0, 0, 0, 0, 0, 0, 0, 0, 0, 0, 0, 0, 0, 0, 0, 0, 0, 0, 0, 0, 0, 0, 0, 0, 0, 0, 0, 0, 0, 0, 0, 0, 0, 0, 0, 0, 0, 0, 0, 'end']	Paper1: Mexico Paper2: Egypt

## Summary and Future Work

This article described the methods implemented to extract the functional unit, the impact characterization method, and the case study location of the LCAs considered for a literature review. Although these characteristics were mined from wastewater-based resource recovery LCAs, the methods can be employed to evaluate other types of engineered systems and extract alternative types of data such as the goal of the study, the life cycle phases considered, and so on. Future work is recommended in developing a method to automatically extract the information from the journal article in its HTML form based on the web address. This would further automate the data extraction process and eliminate the need to transfer the article’s content to text files.

## Declaration of Competing Interest

The authors declare that they have no known competing financial interests or personal relationships that could have appeared to influence the work reported in this paper.

## Appendix Supplementary materials

Image, application 1
